# Suicidal Behavior in a Patient with Burning Mouth Syndrome

**DOI:** 10.1155/2014/405106

**Published:** 2014-08-26

**Authors:** Konstantinos Kontoangelos, Evmorfia Koukia, Vasilis Papanikolaou, Aris Chrysovergis, Antonis Maillis, George N. Papadimitriou

**Affiliations:** ^1^1st Department of Psychiatry, Athens University Medical School, Eginition Hospital, 115 28 Athens, Greece; ^2^University Research Institute of Mental Health, 11527 Athens, Greece; ^3^Department of Mental Health and Behavioral Sciences, Faculty of Nursing, University of Athens, 11527 Athens, Greece; ^4^1st University Department of Otorhinolaryngology, Head and Neck Surgery, Hippokration Hospital, Athens University, 11527 Athens, Greece

## Abstract

*Introduction*. Chronic pain of the oral cavity is a long-term condition and like all other types of chronic pain is associated with numerous comorbidities such as depression or anxiety.* Case Presentation*. This is a case of a 93-year-old patient suffering from chronic oral cavity pain who repeatedly stabbed his palate due to ongoing local pain, over the last few months, which he could not further tolerate. The patient was suffering from depression and also a diagnosis of “burning mouth syndrome” (BMS) was made. *Discussion*. Burning mouth syndrome (BMS) is characterized by a burning sensation in the tongue or other oral sites. BMS has high psychiatric comorbidity but can occur in the absence of psychiatric diagnosis. Patients with multiple forms of pain must be considered as potential candidates for underdiagnosed depression (major) and suicidal thoughts.

## 1. Introduction

The multidimensionality of chronic pain of the oral cavity is connected with psychosocial factors. Many patients will have more than one pain diagnosis and there may also be an underlying psychiatric or personality disorder which predisposes to chronic pain and which may modify clinical course that often leads to false management [1]. Chronic oral pain is related with posttraumatic trigeminal neuropathy, trigeminal neuralgia, persistent idiopathic facial pain, trigeminal postherpetic neuralgia, temporomandibular disorders, and burning mouth syndrome [2]. Burning mouth syndrome has been defined as burning pain in the tongue or oral mucous membranes, usually without accompanying clinical and laboratory findings. Pain is absent during the night but occurs at a mild to moderate level by middle to late morning. Patients often report that the pain interferes with their ability to fall asleep. Sleep disorder and constant pain alone or together coexist with disturbances, that is, irritability, anxiety, and depression. Epidemiological studies on BMS have shown a prevalence of 2.6% to 5.1% and that the ratio of occurrence of the disorder in men is less than 20% of that in women [3]. Concerning the etiology of BMS, it has been suggested that the etiological factors can be divided into three main groups: local, systemic, and psychogenic [4]. In the genesis of BMS, the possible role of stressful life events and of long-term social problems and also that 50% of the cases of BMS are comorbid with other current psychiatric disorders. Depressive symptoms (major depressive episode and dysthymic disorder) represent the most prevalent concurrent (32.3%) and lifetime (36.3%) codiagnoses. In most cases after the first episode of BMS a major depressive episode alone or together with GAD follows, suggesting a precipitating role in the onset of these disorders [5]. We present a case of a patient suffering from chronic oral pain who repeatedly stabbed his palate due to ongoing local pain, over the last few months, which he could not any more tolerate.

## 2. Case Presentation

A 93-year-old male was admitted to the 1st University Department of Otorhinolaryngology via the Accident and Emergency Department due to trauma to the oral cavity. He was found unconscious, by his relatives, bleeding from the oral cavity, and with a blood stained knife in his hand. He was urgently transferred to the hospital, where he received the appropriate resuscitation treatment. Examination of the oral cavity revealed extensive trauma to the soft and hard palate, without any other lesions to be seen in the head and neck region. Following stabilization of his condition the patient reported that he repeatedly stabbed his palate due to the intolerable character of the ongoing local pain, over the last few months, which he could not tolerate. He wanted to die from bleeding because he could not eat and he felt helpless. He preferred to die quickly using a knife instead of dying of thirst and hunger. Prior to the incident he had chronic limited mobility due to osteoarthritic problems and he spent most of the day confined to bed. Further history retrieved from the patient and his family revealed that over a period of approximately two months prior to the event the patient reported a painful oral cavity. He had a burning sensation in the tongue, and he was complaining of oral sensory discomfort, including dryness and taste alterations. The clinical symptomatology had led to a decrease in oral consumption of food and liquids, causing a significant weight loss of more than 10 kg during the last two months. Despite various visits and treatments by the family physician the condition had not improved and the emotional condition of the patient had progressively deteriorated. On admission to the Department of Otorhinolaryngology, Head and Neck Surgery, a psychiatric evaluation following his recovery revealed depressed mood most of the day, marked diminished interest or pleasure in all or almost all activities, insomnia, and fatigue with diminished ability to think or concentrate, and the Beck Depression Inventory (BDI) [6] showed a score of 24. During the clinical assessment, the patient had a mini-mental score of 29 with no delusions and the diagnosis of cenesthopathic schizophrenia was excluded. Regarding somatoform disorders, pain in the oral cavity started the last three months, only in this specific anatomic area, and the patient had no gastrointestinal or sexual or pseudoneurological symptoms. Paroxetine was gradually administered, with a starting dose of 20 mg once a day that over a period of three weeks increased to 40 mg a day. During the following days, the traumatized area presented normal healing with no obvious abnormalities. Further clinical examination of the oral cavity and pharynx, biopsies from the soft and hard palate, and appropriate imaging did not reveal any other pathologies. On discharge, 3 weeks later, he scored 14 on the BDI. The patient reported on his last assessment, one month later, that he could now enjoy his daily activities and be more active and less fearful of his pain symptoms that now were milder after the antidepressant treatment while suicidal ideation had fully resolved.

## 3. Discussion

Based on the patient's age, the lack of any obvious findings, and the specific characteristics of the reported oral pain, a diagnosis of “burning mouth syndrome” was made. This syndrome can be defined as a burning sensation of the intraoral mucosa, with no identifiable medical or dental cause. The reported frequency varies from 3.7% to 40%, being more prevalent in the elderly [7]. The etiology of this condition is poorly understood, and there are no specific diagnostic criteria or therapeutic approaches. Due to this lack of appropriate clinical data and treatment, the condition is usually inefficiently managed, causing a significant decrease of the quality of life and a continuous somatic and psychological stress [8]. Personality and mood changes have been consistently demonstrated in patients with burning mouth syndrome suggesting that these conditions are closely related to psychological problems. However, psychological dysfunction is common in patients with chronic pain either as triggering modalities or as itself [9]. Individuals with chronic pain have a higher prevalence of depression, anxiety, alcohol and drug abuse, or dependence than those without pain. Prior research has identified a link between pain and suicide. In a recent review, it was reported that chronic pain was associated with increased risk of suicide mortality and that the rates of suicidal ideation were higher in individuals with pain than those without [10, 11]. Edwards and colleagues found that over 30% of patients seeking treatment for chronic pain reported some form of recent suicidal ideation [12].

Although existing initiatives for reducing suicide risk emphasize the importance of examining several demographic, psychiatric, environmental, and physical health-related risk factors for suicide, in practice, many efforts to identify and target at-risk patients focus only on the presence of psychiatric symptoms or recent suicide attempts [13]. Any levels of pain in home care elders should be taken seriously and treated as one means to reduce risk of suicide. Prior research has reported that undertreatment of pain is pervasive in older persons and those who are older or cognitively impaired are more likely to receive unsatisfactory analgesia. While interventions at multiple levels are needed, public policies could play a role in improving pain management [14].

Those with multiple forms of cooccurring pain must be considered as potential candidates for underdiagnosed depression (major) and suicidal thoughts. This must never be underestimated particularly among elderly incapacitated patients living alone and suffering from chronic undertreated pain or depression.
